# A potential association of *RNF219*‐*AS1* with ADHD: Evidence from categorical analysis of clinical phenotypes and from quantitative exploration of executive function and white matter microstructure endophenotypes

**DOI:** 10.1111/cns.13629

**Published:** 2021-02-28

**Authors:** Guang‐Hui Fu, Wai Chen, Hai‐Mei Li, Yu‐Feng Wang, Lu Liu, Qiu‐Jin Qian

**Affiliations:** ^1^ Peking University Sixth Hospital/Institute of Mental Health Beijing China; ^2^ National Clinical Research Center for Mental Disorders & The Key Laboratory of Mental Health Ministry of Health (Peking University Beijing China; ^3^ Mental Health Service Fiona Stanley Hospital Perth Australia; ^4^ Graduate School of Education The University of Western Australia Perth Australia; ^5^ School of Medicine The University of Notre Dame Australia Fremantle Australia; ^6^ School of Psychology Murdoch University Perth Australia

**Keywords:** ADHD, inhibition, lncRNA, *RNF219‐AS1*, white matter microstructure

## Abstract

**Aims:**

Attention‐deficit/hyperactivity disorder (ADHD) is a neuropsychiatric disorder of substantial heritability, yet emerging evidence suggests that key risk variants might reside in the noncoding regions of the genome. Our study explored the association of lncRNAs (long noncoding RNAs) with ADHD as represented at three different phenotypic levels guided by the Research Domain Criteria (RDoC) framework: (i) ADHD caseness and symptom dimension, (ii) executive functions as functional endophenotype, and (iii) potential genetic influence on white matter architecture as brain structural endophenotype.

**Methods:**

Genotype data of 107 tag single nucleotide polymorphisms (SNP) from 10 candidate lncRNAs were analyzed in 1040 children with ADHD and 630 controls of Chinese Han descent. Executive functions including inhibition and set‐shifting were assessed by STROOP and trail making tests, respectively. Imaging genetic analyses were performed in a subgroup of 33 children with ADHD and 55 controls using fractional anisotropy (FA).

**Results:**

One SNP rs3908461 polymorphism in *RNF219*‐*AS1* was found to be significantly associated with ADHD caseness: with C‐allele detected as the risk genotype in the allelic model (*P* = 8.607E‐05) and dominant genotypic model (*P* = 9.628E‐05). Nominal genotypic effects on inhibition (*p* = 0.020) and set‐shifting (*p* = 0.046) were detected. While no direct effect on ADHD core symptoms was detected, mediation analysis suggested that SNP rs3908461 potentially exerted an indirect effect through inhibition function [B = 0.21 (SE = 0.12), 95% CI = 0.02‐0.49]. Imaging genetic analyses detected significant associations between rs3908461 genotypes and FA values in corpus callosum, left superior longitudinal fasciculus, left posterior limb of internal capsule, left posterior thalamic radiate (include optic radiation), and the left anterior corona radiate (*P*_FWE corrected_ < 0.05).

**Conclusion:**

Our present study examined the potential roles of lncRNA in genetic etiological of ADHD and provided preliminary evidence in support of the potential *RNF219*‐*AS1* involvement in the pathophysiology of ADHD in line with the RDoC framework.

## Graphical abstract

A key noncoding RNA variant was found to be associated with the risk of ADHD children in Chinese Han, and the mechanism of this variant affected symptom performance was through the indirect effect of inhibition control. This important DNA polymorphism locus in lncRNA could affect white matter microstructure by the imaging genetic analyses.

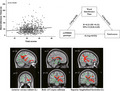

## 1 INTRODUCTION

Attention‐deficit/hyperactivity disorder (ADHD) is a neurodevelopmental disorder characterized by age‐inappropriate levels of inattentive, hyperactive, or impulsive symptoms, with a worldwide childhood prevalence around 5%.^1^ Quantitative genetic studies identity a high heritability in ADHD, estimated to be around 76%^2^; however, as a complex polygenic disorder, its precise genetic basis remained elusive.

Genome‐wide association studies (GWAS), a powerful research tool, can screen a large number of genetic markers for disorder phenotypes and complex traits^3^ and have demonstrated that the majority (~93%) of risk‐associated single nucleotide polymorphisms (SNP) were located in the noncoding regions.^4^ For ADHD, in line with other GWAS findings, most of its risk variants (~90%) were also found in the 3‐untranslated region (3’UTR), 5ʹ flanking, intron, and other noncoding regions instead of the coding regions of genome.^5^ The Research Domain Criteria (RDoC) approach, proposed by National Institute of Mental Health, emphasizes the importance of shifting research focus from diagnostic categories of mental disorders to the multiple level analytic approach that integrate genes, molecules, brain circuitry, cognitive performances, and behaviors.^6^ Notably, intermediate phenotypes or endophenotypes are hypothesized as more informative characteristics for probing genetic and molecular substrates. In the post‐GWAS era, we highlighted the importance in focusing in the noncoding regions, in particular candidate variants informed by our literature review on animal studies and ADHD GWAS studies. Of these, we here selected the long noncoding RNAs (lncRNAs) as our target genetic candidate from the noncoding region; and we proposed to examine their potential roles in ADHD pathophysiology by adapting the RDoC approach. More specifically, in this study, we explored the association of lncRNAs with ADHD, which we chose to be represented at three different phenotypic levels as guided by the RDoC: (i) at the first level, ADHD caseness and symptom dimension, (ii) at the second, executive functions as functional endophenotype and (iii) finally, potential genetic influence on white matter architecture as brain structural endophenotype.

Indeed, it has been well‐established that the polymorphisms and allelic variation of noncoding regions of the genome influences gene expression; and these variations—scattered throughout the whole genome—are more common than polymorphisms in the coding regions in influencing disorder risks.^7^ More specifically, these variants play critical roles in the cis‐regulation of nearby genes or trans‐regulation of distant genes at both transcriptional and post‐transcriptional levels. The risk‐associated noncoding SNPs identified by GWAS studies include variants in promoters, enhancers, transcription factor (TF) binding sites, and other important regions associated with components of critical signaling pathways.^4^ Moreover, specific molecular mechanisms have been revealed by systematic research for neurodevelopmental and neuropsychiatric disorders; and these include regulative loci (enriched in the noncoding regions) which could fine‐tune the splicing process of transcription product and interact with other genes and proteins within the molecular networks in the pathophysiological pathways for ADHD (such as synaptic transmission, catecholamine metabolic process, G‐protein signaling, coupled to cyclic nucleotide second messenger, learning or memory, and cell migration).^8^ Therefore, noncoding SNPs may represent novel fruitful avenues to explore etiological factors in ADHD.

In the last decade, the Encyclopedia of DNA Elements (ENCODE) project has systematically sequenced the whole human genome. Strikingly, only 2.94% of genomic sequences were classified as protein‐coding genes (ie, GENCODE‐annotated exons), with 97% of DNA sequences found in the noncoding regions.^9^ Long noncoding RNAs (lncRNAs) is a specific class of RNA with the length more than 200 nucleotides and lacked protein‐coding potential; and these sequences cover about 27% of non‐protein‐coding transcripts in the human genomic annotation.^10^ The majority of lncRNAs were expressed uniquely in the brain with the spatial and temporal specificity; and their levels undergo dynamical changes during the development.^11^ Critically, lncRNAs participated in the process of maintaining the differentiation potential of brain neural progenitor cells (NPCs), synaptogenesis and cell‐specific differentiation all of which are critical in epigenetic regulation of gene expression^12^; as such, lncRNAs have been proposed as candidate substrates in the pathophysiology of other neuropsychiatric disorders such as autism spectrum disorder (ASD), schizophrenia (SZ), major depressive disorder (MDD), and substance dependence.

Notably, the largest GWAS meta‐analysis to date performed in ADHD has reported 12 genome‐wide significant loci (*p* < 5E‐08), which span several long noncoding RNA sequences, such as *KDM4A*‐*AS1*,*LINC02497*,*LINC02060*,*TMEM161B*‐*AS1*,*LINC01288*,*LINC01572*,*MEF2C*‐*AS1*,*and LINC00461*.^13^ Of particular interest is the intergenic lncRNA *LINC00461*, which showed the highest pleiotropic effects involved in five psychiatric traits: SZ, ADHD, depression, neuroticism, and anxiety disorder.^14^ Interestingly, the index variant rs12661753 located in the long noncoding RNA gene *STXBP5*‐*AS1* was found to be associated with ADHD symptom scores in another GWAS meta‐analysis consisted of adult population‐based and case‐only cohorts of adults (*p* = 3.02E‐7). The gene‐based analysis showed that three lncRNAs (*STXBP5*‐*AS1*, *LINC01247*, and *LINC00534*) were also associated with ADHD symptoms (*p* < 0.007).^15^ One SNP rs4404327 which located in a neuron‐specific lncRNA (*BC200 RNA*) was also found to be associated with ADHD in an Iranian population.^16^ However, none of the above analyses have adopted the RDoC approach to probe beyond the ADHD clinical phenotypes, such as on executive function and brain structural integrity as potential endophenotypes more proximal to gene‐molecule mechanisms.

ADHD is currently conceptualized as a neurodevelopmental disorder characterized by multi‐dimensional symptoms, which represent the extreme ends of quantitative behavioral traits, continuous with the general population; and ADHD is therefore not the expression of a categorical or discrete disease state.^17^ The pathogenesis of ADHD is considered as multi‐factorial, and genetic variants might affect multiple dimensional traits such as clinical symptoms, neuropsychological performance, and brain structural/functional features.^18^ Recently, advances in behavioral genetics and neuroimaging genetics have provided important theoretical perspectives for understanding the pathophysiological changes of ADHD, which can be mapped onto the biological pathways from gene to disease. Here, we posit that the RDoC framework applied to ADHD at the cognition and neurocircuitry levels would provide additional advantages for research initiatives.^19^ Executive function (EF) refers to task‐oriented cognitive processes which require deliberate and effortful control that involves inhibition, working memory, monitoring, and execution.^20^ Inhibition and set‐shifting—two core processes of EF—are informative cognitive endophenotype of ADHD.^21^, ^22^, ^23^, ^24^ The brain tissue‐expressed lncRNAs have been found to carry out important functions in brain development, maintenance of neural cell function, pluripotency, and neuronal differentiation,^25^ which are relevant to executive functions. Further support for the critical roles of lncRNAs in neural integrity has been shown in animal model. In a rodent ADHD model, one recent study identified a large number of dysregulated expression of lncRNAs in the hippocampus of the brains of the affected rats, and these lncRNAs were involved in the biological pathway of brain developmental processes and neuronal function and maintenance.^26^ Overall, different strands of emerging evidence from GWAS and animal studies converge to indicate that the risk variants of lncRNAs can detrimentally affect brain development, structure, function, and cognition; and these neural substrates represent potential candidate targets in the exploration of the underlying neuropathological mechanisms of ADHD.

To further elucidate the potential role of lncRNA in the etiology of ADHD, our study first conducted a categorical case‐control association analyses on the clinical phenotype of ADHD in Chinese Han subjects. For the identified risk loci, quantitative trait loci (QTL) analyses were further conducted to explore their genetic effects on ADHD core symptoms and executive functions. Thereafter, imaging analyses in a subgroup were performed to investigate the potential underlying brain mechanisms of ADHD‐related lncRNA variants.

## 2 MATERIALS AND METHODS

### 2.1 Participants and diagnosed

We recruited 1670 subjects (aged between 6 and 16 years old) in the study. A total of 1040 children suffering from ADHD (360 with ADHD inattentive subtype, 680 with ADHD combined subtype) were enrolled from the child psychiatric clinics at Peking University Sixth Hospital/Institute of Mental Health. Psychiatric diagnoses of ADHD and comorbidities were assessed and classified basing on the Diagnostic and Statistical Manual of Mental Disorders, 4th Edition (DSM‐IV), meanwhile quantitative measurement of inattention and hyperactivity/impulsivity symptoms was evaluated by the ADHD rating scale‐IV (ADHD RS‐IV).^27^ A Chinese Wechsler Intelligence Scale for Children^28^ was used to evaluate intelligence quotient (IQ); and those with IQs more than 70 were included. Six hundred and thirty healthy children were recruited as controls from local elementary schools. Both biological parents of all participants were of Chinese Han descent. Exclusion criteria for the both groups were severe physical illnesses, family history of psychiatric disorders, seizure, major depression disorder, panic disorder, psychosis, bipolar disorder, and pervasive development disorders. Controls with a history of the ADHD diagnosis were also excluded. Among 1040 children with ADHD, 806 subjects with available data of both ADHD core symptoms and executive functions were included for the subsequent “Gene‐Behavior/Cognition Analysis.”

For the brain structure study, a subgroup of 33 children with ADHD and 55 healthy controls were included for the magnetic resonance imaging (MRI) genetic analyses. More stringent inclusion and exclusion criteria were applied in order to control for potential confounders. Additional exclusion criteria were as follows: (i) post‐traumatic stress disorder, (ii) history of severe head injury or brain trauma, (iii) claustrophobia, and (iv) having metal implants or other conditions which are contraindication for MRI scanning. More stringent inclusion criteria included the following: (i) right hand dominant and (ii) without history of taking any psychoactive or antipsychotic drug. Signed informed consent was obtained from the parents, and verbal consent was obtained from the children before scanning. These MRI data were partial from the study of Jin et al.^29^ This study was approved by the Ethics Committee of Peking University Sixth Hospital/Institute of Mental Health.

### 2.2 Cognitive function test

#### 2.2.1 STROOP color‐word interference test

The test included four parts consisted of Color Naming (Part 1, naming blocks of color), Word Reading (Part 2, reading colored words printed in black ink), Color Inhibition (Part 3, reading colored words printed in different colors), and Word Inhibition (Part 4, naming the color of words printed in incongruent colors). The “color interference time” is defined as the time spent on Part 3 minus that on Part 2. The “word interference time” is defined as the time spent on Part 4 minus that on Part 1. The Color and Word Interference Time scores were used to evaluate the “Inhibition” function.^30^

#### 2.2.2 Trail making test (TMT)

TMT was used to assess set‐shifting. It includes two sections: number sequencing trail making and number‐letter switching trail making. The time spent on each section was recorded, and the set‐shifting time was represented by the discrepancy in time between “Number‐Letter Switching trail making” and “Number Sequencing trail making.”^30^

### 2.3 LncRNA selection and SNP genotype

We choose candidate ten long noncoding RNAs to evaluate their associations with ADHD risk in our Chinese Han sample: *KDM4A*‐*AS1*,*LINC02497*,*LINC00461*,*LINC02060*,*TMEM161B*‐*AS1*,*LINC01288*,*LINC01572*,*MEF2C*‐*AS1*,*LOC105379109*, and *RNF219*‐*AS1*. These lncRNAs were all from the GWAS‐meta study of Demontis et al^13^ which have shown eight lncRNAs located within 50 kb of the credible set for the significant 12 loci, including *KDM4A*‐*AS1*,*LINC02497*,*LINC00461*,*LINC02060*,*TMEM161B*‐*AS1*,*LINC01288*,*LINC01572*, *and MEF2C*‐*AS1*. Additionally, novel index variants from two lncRNAs (rs1592757/rs30266 in *LOC105379109* and rs2243638/rs9574218 in *RNF219*‐*AS1*, respectively) emerged in the replication GWAS‐meta analysis of the primary ADHD GWAS along with three other independent ADHD‐related GWASs.

All subjects of our study were from the Han Chinese ADHD GWAS project.^31^ We extracted the data for children samples (1040 children with ADHD and 630 healthy controls) for genetic association analysis. First, 501 SNP markers for the above 10 lncRNA genes were obtained from the Affymetrix 6.0 SNP array at Capital Bio Ltd. (Beijing). Then, Haploview v.4.2 (http://www.broad.mit.edu/mpg/haploview/) was used to filter the SNPs with minor allele frequency (MAF) <0.05, a call rate <95%, failing the Hardy‐Weinberg equilibrium (HWE) test (*p* < 0.001), and yielding 328 SNPs. Finally, 107 tag‐SNPs (Table S1) were generated from these 328 SNPs for analysis according to a screening standard of tagging *r* ^2^> 0.8.

### 2.4 Diffusion tensor imaging (DTI) data acquisition and processing

MRI scanning from a subgroup of 33 ADHD and 55 health controls was carried out with a 3.0 T Siemens Tim Trio MRI scanner, using a standard 12‐channel head coil in the Imaging Center for Brain Research, Beijing Normal University. DTI scanning was conducted with the following scan parameters: field of view: 230 × 230 mm, flip angle = 90°, matrix = 128 × 128, slice thickness: 2.5 mm, repetition time [TR]/echo time [TE] =7200/104 ms, 64 optimal nonlinear diffusion‐weighted directions with b = 1000 s/mm^2^ and one additional image without diffusion weighting (ie, b = 0 s/mm^2^), 1.8 × 1.8 mm in‐plane resolution.

For preprocessing the DTI, all DICOM images were transferred to 4D‐Nifti file using the MRIcroN software. Original FA images were created by the following 3 steps using FMRIB Software Library (FSL) version 6.1 (www.fmrib.ox.ac.uk/fsl): (i) head motion and eddy current distortions were corrected by the “eddy” tool in the FMRIB’s Diffusion Toolbox (part of FSL); (ii) Brain Extraction Tool (BET)^32^ was used for brain extraction; and (iii) FA images were obtained by DTIFIT from the FMRIB’s Diffusion Toolbox. Then, tract‐based spatial statistical analysis (TBSS) (http://fsl.fmrib.ox.ac.uk/fsl/fslwiki/TBSS) was applied for voxel‐wise statistics analysis.^33^ All individual FA images were mapped to a standard space based on nonlinear registration. Because of lacking valid template for children, we identified the most representative FA image as the target template through aligning each FA image with others within the whole samples. Finally, the mean FA image and mean FA skeleton were created, and individual FA images were projected onto the mean FA skeleton while the resulting data were fed into voxel‐wise statistics.

### 2.5 Statistics analyses

#### 2.5.1 Demographic and clinical characteristics

Statistical analyses of demographic and clinical characteristics, with chi‐square test for categorical variable (sex) and *t* test for continuous variables (age, IQ) were carried out by SPSS v22.0 software. Before statistical analysis, the normality of continuous variables (age, IQ) was tested through analysis of skewness and kurtosis, with acceptable scores between −1 and 1.

#### 2.5.2 Gene‐behavior/cognition analysis

Chi‐square tests were used to examine the allelic distributions of the candidate SNPs between ADHD and health controls. When the allelic model difference was nominally significance (*p* < 0.05), further analyses under additive, recessive, and dominant models were also conducted. For the SNPs showing significant allelic and/or genotypic association with ADHD, analysis of covariance (ANCOVA) was further conducted to test its influence on ADHD core symptoms and cognitive functions, while controlling for gender, age, and IQ as potential confounders. All continuous variables (scores of ADHD core symptoms and cognitive functions) were first checked for normality of data distribution with acceptable scores of skewness and kurtosis between 1 and −1. Levene's test for the homogeneity of variances (all *p *> 0.05) was performed prior to the ANCOVA analysis. Bonferroni corrections were performed to correct for multiple comparisons, setting the significant *P*‐value at 1.168E‐04 for the categorical genetic analyses (0.05/107/4, where 107 represents the number of SNPs analyzed, 4 represents the allelic and genotypic models) and 0.008 for the quantitative genetic association analyses (0.05/6, 6 represents the ADHD core symptoms and executive functions). Statistical analyses were performed with SPSS v22.0 software (Inc., Chicago, IL, USA) and Haploview version 4.2. (http://www.broad.mit.edu/mpg/haploview/).

Previous studies suggested that impaired cognitive function was related to the ADHD core symptoms, and GWAS of cognitive‐behavioral phenotypes have provided important information about the genetic basis of endophenotypes.^34^ Inhibition and set‐shifting ability, as intermediate phenotypes between genes and phenotypes, were heritable^35^ and maybe involved in the pathological pathways from ADHD‐associated genes to the core symptoms.^36^ Therefore, in this study, the association analyses of ADHD symptom scores and cognitive function scores were conducted by Pearson correlation analysis, and mediation analyses were further performed to explore the cognitive mediation effects between genotype and clinical symptom scores by using PROCESS.^37^

#### 2.5.3 Functional annotations of significant SNPs

The functional information for significant SNPs was retrieved from the public bioinformatics resources. We detected the effects of positive SNPs on gene expression levels by carrying out expression quantitative trait loci (eQTL) analysis based on BRAINEAC database (https://caprica.genetics.kcl.ac.uk/BRAINEAC/) generated from the UK Brain Expression Consortium (UKBEC).

#### 2.5.4 Imaging genetic analysis

Given the findings from the eQTL analysis and our previous findings that white matters alterations existed in many brain regions of ADHD children compared to health children,^38^ our present study further aimed to explore the possible genetic effects of the observed ADHD‐related lncRNA variants on the altered white matter microstructure in children with ADHD. The genotype of 33 ADHD and 55 healthy children was redistributed into two groups based on risk‐allele genotypes in the analyses (for rs3908461, as the sample was divided into “TC+CC” genotype versus “TT” genotype groups). After checking the FA values for normality and the data passing Levene's test for equality of variances (*p* > 0.05), comparison of FA values between the two groups was performed using analysis of variance (ANCOVA) within the framework of the general linear model (GLM) in a whole‐skeleton voxel‐based manner with TBSS, adjusting for gender, age, IQ, and ADHD diagnosis variables. Threshold‐free cluster enhancement (TFCE) was applied to obtain cluster‐wise statistics corrected for multiple comparisons. For controlling the family‐wise error (FWE), the data were null permuted 5000 times to identify the clusters at a threshold level of *p* < 0.05.

JHU DTI‐based white matter atlases in FSL were used to annotate the clusters’ position, the ICBM‐DTI‐81 white matter labels atlas provided by Laboratory of Brain Anatomical MRI (Johns Hopkins University). To further explore whether the white matter alterations have an effect on the cognitive functions, the mean FA values within each significant cluster matching to these atlases were extracted and entered into partial correlation analyses with the cognitive functional scores, after adjusting for age, gender, and IQ. The analyses were performed separately in the ADHD and control groups.

## 3 RESULTS

Of 1670 participants (1040 ADHD children and 630 controls) examined in this study, there was no significant difference of age between the two groups, but the IQ of children with ADHD was lower than the controls, with higher percentage of male children in the ADHD group. The same patterns were also observed in the imaging genetic analyses, and the details were summarized in Table 1.

**TABLE 1:** Demographic and clinical characteristics of the children with ADHD and healthy controls

	Gene diagnosis				Gene‐behavior/cognition	Imaging genetic analysis			
	ADHD (n = 1040)	Control (n = 630)	*χ* ^2^ */t*	*P*	ADHD (n = 806)	ADHD (n = 33)	Control (n = 55)	*χ* ^2^ */t*	*P*
Age [Mean (SD)]	9.70 (2.46)	9.53 (1.79)	1.46	0.145	10.07 (2.35)	10.64 (1.79)	10.22 (1.72)	1.08	0.281
IQ [Mean (SD)]	103.92 (14.71)	112.88 (14.09)	9.36	**<0.001**	104.65 (14.64)	106.82 (14.88)	115.55 (13.06)	2.85	**0.005**
Male [n (%)]	876 (84.23)	353 (56.30)	157.54	**<0.001**	688 (85.40)	30 (90.90)	25 (45.45)	18.18	**<0.001**
ADHD subtype [n (%)]									
ADHD‐I	360 (34.60)	—			297 (36.80)	12 (36.4)	—		
ADHD‐C	680 (65.40)	—			509 (63.20)	21 (63.6)	—		

Abbreviations: ADHD, attention‐deficit/hyperactivity disorder; ADHD‐C, ADHD combined subtype; ADHD‐I, ADHD inattentive subtype; IQ, intelligence quotient; SD, standard deviation.

### 3.1 Gene‐behavior/cognition analysis

Significant associations between the SNP rs3908461 of *RNF219*‐*AS1* and ADHD were detected under the allelic model [*P* =8.607E‐05; OR = 1.35, 95% confidence interval (CI) 1.16‐1.58)] and dominant genotypic model [*P* =9.628E‐05; OR = 2.00, 95% CI (1.41‐2.86)], with C‐allele as risk factor (Table 2). In addition, the allelic distribution of another 4 SNPs (rs9935250 in *LINC01572*; rs71106003, rs10507880, and rs9600980 in *RNF219*‐*AS1*) were also different between ADHD children and controls at the nominal significance levels of *p* < 0.05.

**TABLE 2:** Association between candidate SNPs and ADHD

Gene symbol	SNP ID	A1	A2	Allelic comparison			Additive model		Dominant model		Recessive model	
				A1/A2 (case: control)	OR (95% CI)	P*	A1A1/A1A2/ (case: control)	P*	OR (95% CI)	P*	OR (95% CI)	P*
*LINC01572*	rs9935250	**T**	C	1618/462: 935/325	1.22 (1.03‐1.43)	0.018	633/352/55: 344/247/39	0.042	1.18 (0.78‐1.82)	0.439	1.30 (1.06‐1.59)	0.012
*RNF219‐AS1*	rs3908461	**C**	T	1505/567: 833/425	1.35 (1.16‐1.58)	**8.607E−5**	538/429/69: 283/267/79	2.497 E−4	2.00 (1.41‐2.86)	**9.628E−5**	1.30 (1.05‐1.61)	0.012
	rs71106003	**C**	T	1528/492: 903/353	1.21 (1.04‐1.42)	0.005	594/394/49: 326/251/51	0.005	1.69 (1.14‐2.56)	0.011	1.27 (1.03‐1.54)	0.027
	rs10507880	**T**	C	1656/424: 960/300	1.22 (1.03‐1.44)	0.020	663/330/47: 367/226/37	0.029	1.33 (0.85‐2.08)	0.206	1.25 (1.02‐1.54)	0.038
	rs9600980	G	**C**	847/1227: 469/791	1.16 (1.00‐1.34)	0.038	362/503/172: 255/281/94	0.034	0.88 (0.96‐1.16)	0.366	0.78 (0.63‐0.96)	0.018

Abbreviations: OR, odd ratios; 95% CI, 95% confidence interval. P* the significant level was corrected with the formula of *p* = 0.05/107/4 ≈1.168E‐4 according to the Bonferroni method. The risk alleles and significant results were shown in bold.

Quantitative analyses of ADHD core symptoms and cognitive function scores were conducted in 806 children with ADHD with available and complete data for analyses. The ANCOVA analyses (Table 3) indicated longer Word Interference Time [(30.62 ± 18.01) vs (25.69 ± 11.40), *p* = 0.020)] and Shifting time [(147.85 ± 107.46) vs (119.02 ± 101.44), *p* = 0.046)] in the C‐allele carriers than TT carriers with gender, age, and IQ adjusted; however, those association did not survive Bonferroni corrections for multiple comparisons. No genotypic effects of rs3908461 on ADHD core symptoms were identified (all *p *> 0.05).

**TABLE 3:** The association of rs3908461 with ADHD core symptoms and executive functions

Phenotype	Genotype (n)		*F*	P^*^
	TT (51)	TC +CC (749)		
	Mean ±SD	Mean ±SD		
Core symptoms (ADHD RS‐IV)				
Inattentive scores	18.91 ± 4.10	18.90 ± 3.75	0.02	0.883
Hyperactive‐Impulsive scores	14.59 ± 4.61	14.95 ± 5.26	0.14	0.714
Total scores	33.62 ± 7.75	33.82 ± 7.55	0.01	0.930
Executive function				
STROOP color‐word interference test				
Color interference time	5.96 ± 6.16	7.01 ± 9.87	0.69	0.406
Word interference time	25.69 ± 11.40	30.62 ± 18.01	5.43	**0.020**
Trail making test (TMT)				
Set‐shifting time	119.02 ± 101.44	147.85 ± 107.46	4.00	**0.046**

Abbreviations: ADHD, attention‐deficit/hyperactivity disorder; ADHD RS‐IV, ADHD rating scale‐IV. P* was adjusted with sex, age, and IQ, the significant level was corrected with the formula of *p* = 0.05/6 ≈0.008 according to the Bonferroni method. The nominally significant results were shown in bold.

**FIGURE 1:**
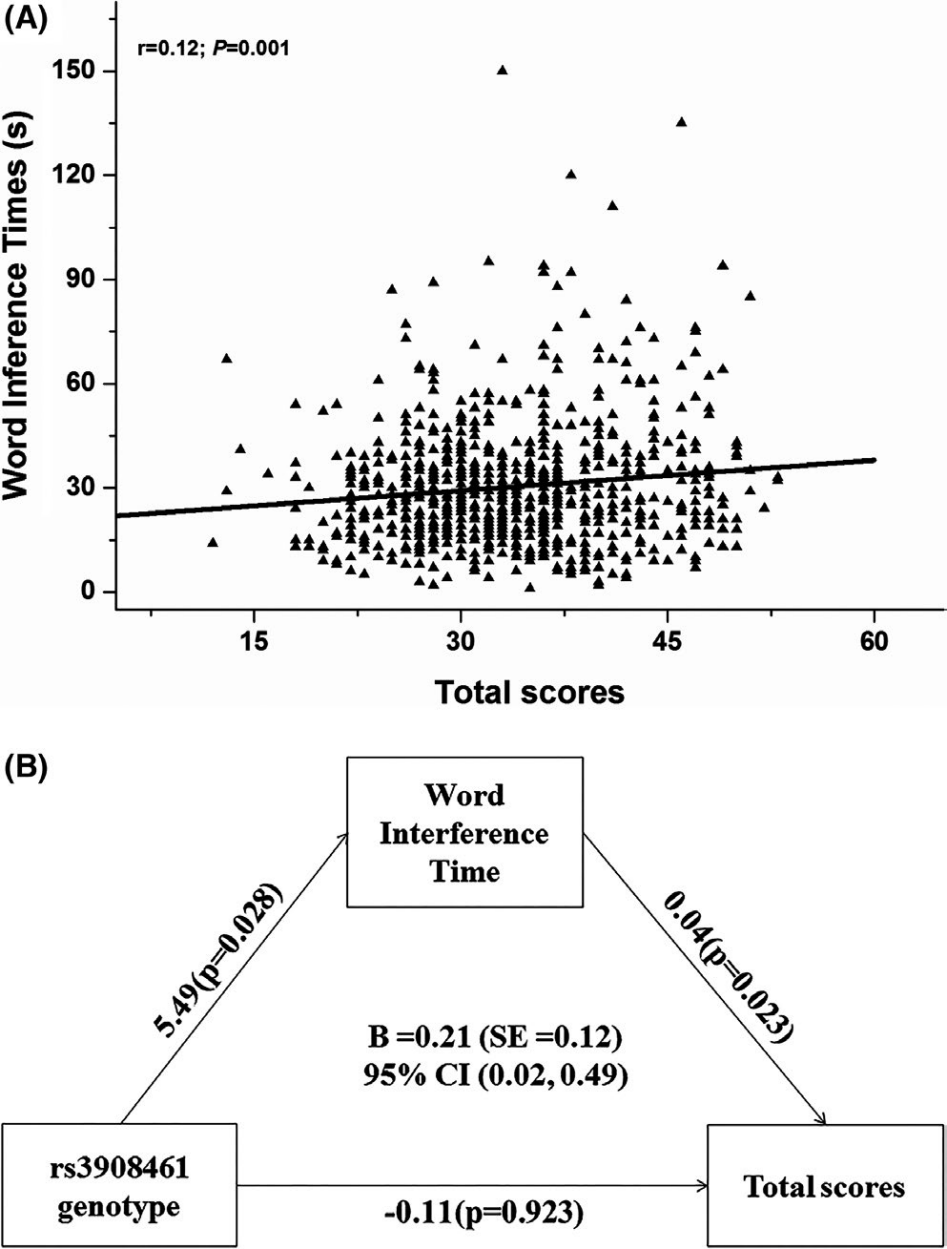
The correlation between ADHD total scores and word interference time in the ADHD group. A, The increased word interference time in STROOP test (indicated the poor inhibition function) was associated with the increases in ADHD total scores. B, The full mediation model of word interference time (inhibition) on the relationship between genotype and ADHD total scores (symptoms)

The mediation analysis for rs3908461 and core symptom with cognitive function scores as the mediator were performed. First, we explored the correlations between the STROOP test (“color interference time” and “word interference time”)/Trail making test (“shifting time”) and ADHD core symptoms (Table S2). The positive correlations of the word interference time with hyperactive/impulsive (*r* = 0.13, *P* = 4.460E‐4) and ADHD total scores (*r* = 0.12, *p* = 0.001) were detected, likewise, the set‐shifting time and hyperactive/impulsive scores (*r* = 0.08, *p* = 0.024). After adjusting for the gender, age, and IQ, only weak correlation between the word interference time and the ADHD total scores remained significant (*r* = 0.08, *p* = 0.022) (Figure 1A). Then, mediation analysis was carried to explore the potential mediation effect of inhibition on rs3908461 genotype and ADHD total symptoms. The result showed that the direct path from genotype to total scores did not reach statistical significance, but the indirect path through word interference time was significant [B =0.21 (SE = 0.12), 95% CI = 0.02‐0.49] (Table S3). A complete mediation model was detected, showing that word interference time mediated the path between genotype and total scores (Figure 1B): this means that the mechanism of SNP rs3908461 effects on ADHD total symptom was accounted by the indirect effect of inhibition control as the mediator.

### 3.2 eQTL analysis for rs3908461

**FIGURE 2:**
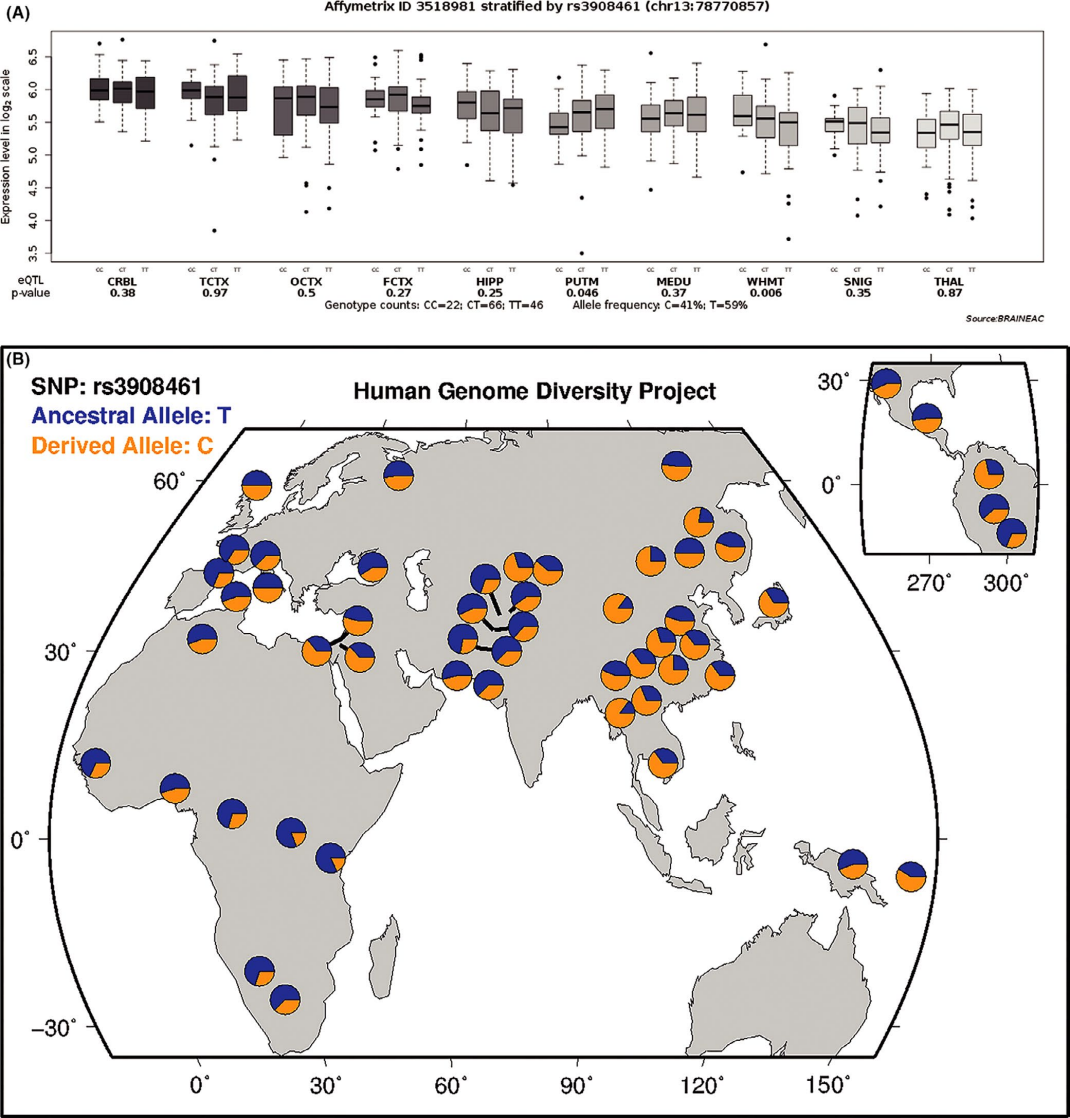
A, eQTL analysis for rs3908461 on *RNF219* transcriptional expression in human brain tissues based on the BRAINEAC database. B, Worldwide diversity of rs3908461 allele frequencies in Human Genome Diversity Project (https://genome.ucsc.edu/trash/hgc). CRBL, cerebella cortex; TCTX, temporal cortex; OCTX, occipital cortex (specifically, primary visual cortex); FCTX, frontal cortex; HIPP, hippocampus; PUTM, putamen; MEDU, medulla (specifically, inferior olivary nucleus); WHMT, intralobular white matter; SNIG, substantia nigra; THAL, thalamus

The eQTL data from the BRAINEAC database showed that the rs3908461 genotype affected *RNF219* mRNA expression in the region of intralobular white matter (*p* = 0.006, Figure 2A), indicating that the minor C‐allele can significantly increase the gene expression level compared to T allele. The UK Brain Expression Cohort was based on Caucasian subjects. When checking the allele frequency in Human Genome Diversity Project, we found that the minor allele in Chinese Han subjects was T, but not C (Figure 2B).

### 3.3 Genotype, white matter microstructure, and inhibition function

To increase statistical power, we examined the effect of C‐genotype rather than ADHD status on white matter structure. First, the effect of C‐genotype on white matter microstructures was detected in the combined group (33 children with ADHD and 55 controls) including 75 C‐allele carriers and 13 TT homozygotes for rs3908461. Whole‐skeleton voxel‐wise statistics analysis based TBSS demonstrated that the C‐allele carriers have the higher FA values than the TT carriers in two clusters, and the significant voxels are particularly located in regions of genu, body, and splenium of corpus callosum, left superior longitudinal fasciculus, left posterior limb of internal capsule, left posterior thalamic radiate (include optic radiation), and the left anterior corona radiate (Table 4).

**TABLE 4:** Effect of rs3908461 genotype on FA in combined samples of ADHD and control

Genotype	Cluster index	White matter tracts^a^	n voxels	MNI coordinate of the peak voxel (x; y; z)	*p*‐value
TC+CC > TT	1	Genu, Body, and Splenium of corpus callosum; superior longitudinal fasciculus (L); posterior limb of internal capsule (L); posterior thalamic radiate(include optic radiation) (L)	15 679	14;−5;34	0.006
	2	Anterior corona radiate (L)	272	−17;14;−18	0.047

^a^ White matter tracts as defined with the JHU ICBM‐DTI‐81 White Matter Labels; The significant clusters with cluster size ≥100 voxels were adjusted with sex, age, IQ and ADHD diagnosis variables, the significant level was at *p* < 0.05 according to the family‐wise error corrected.

**FIGURE 3:**
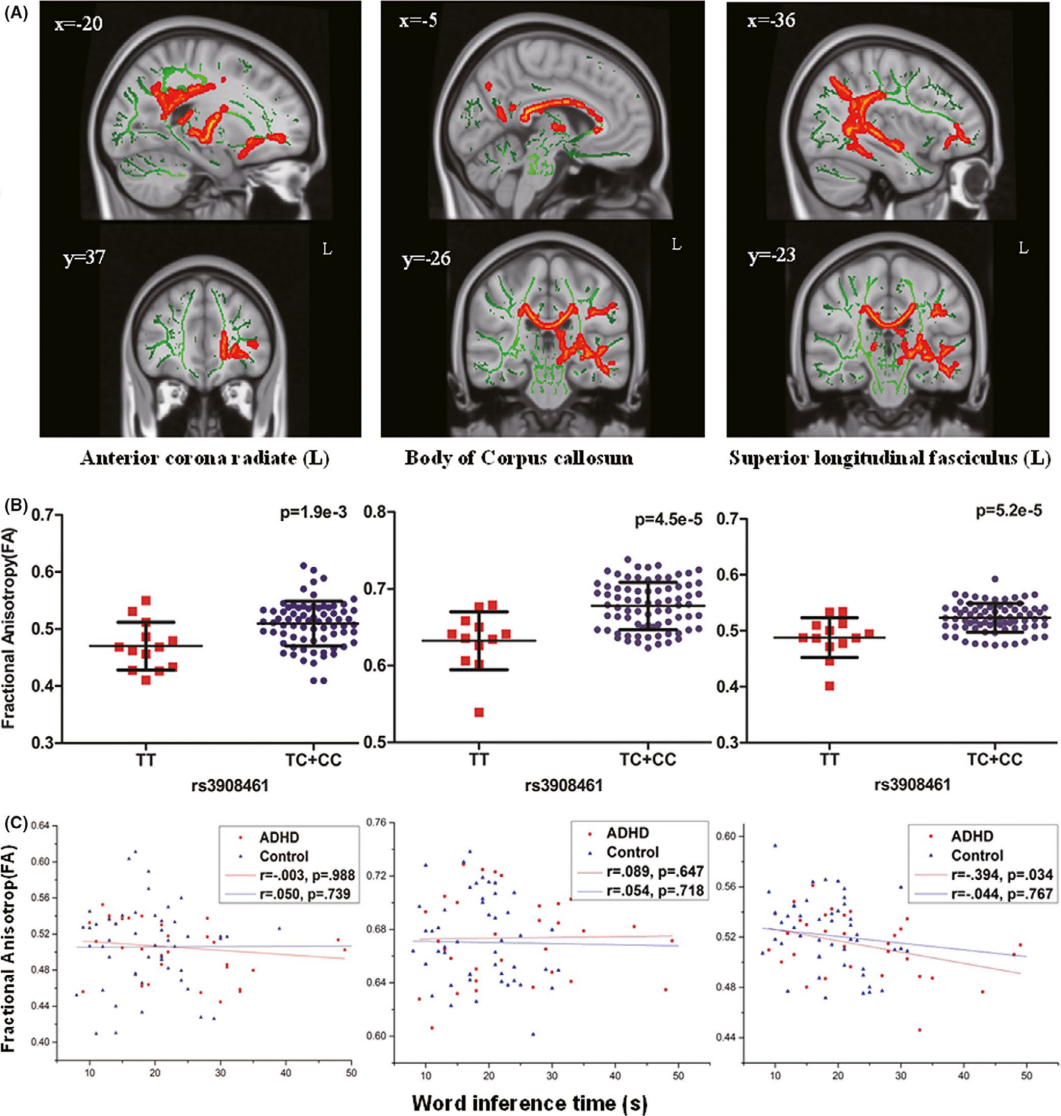
A, Regional differences between two genotypes of rs3908461 on fractional anisotropy (FA) maps. B, Higher FA was associated with rs3908461 risk allele (C) dosage in the combined sample (n = 88) of ADHD participants (n = 33) and controls (n = 55) in three white matter regions. C, The correlation between FA and inhibition function in the ADHD group (the red lines) and the control group (the blue lines) separately in the three brain white matter tracts

Of the above regions, three white matter tracts (left anterior corona radiate, body of corpus callosum, and left superior longitudinal fasciculus) overlapped with our previous findings in which the significant difference of FA values existed between ADHD and healthy controls.^38^ For this reason, these three tracts were selected as candidate regions of interest for further in‐depth analysis. In order to visualize the correlation of genotype of rs3908461 and FA, the FA values from three regions in the significant clusters were extracted and plotted (Figure 3A). General linear model analyses were re‐run for validation separately in the three regions. The results were consistent with the previous TBSS analysis, with higher FA values existed in C‐allele carriers than the TT homozygotes (Figure 3B) (with *P*‐values of 1.9E‐03, 4.5E‐05, and 5.2E‐05, respectively).

Second, we examined the executive function association with the structural integrity of these regions, instead of just the genotype effect. In the aforementioned three regions, we explored the correlations between the FA values and executive inhibition functions. As shown in Figure 3C, negative correlation of the word interference time with FA values was only found in the region of left superior longitudinal fasciculus (*r* = −0.39, *p* = 0.034) in the ADHD group, while longer word interference time (means worse inhibitory) was linked to decreased FA.

## 4 DISCUSSION

There are three key findings in our study, and the associations of lncRNAs with ADHD were detected at three different phenotypic levels. First, significant associations between the SNP rs3908461 of *RNF219*‐*AS1* and ADHD were detected under the allelic model and dominant genotypic model, with C‐allele as risk factor. However, no direct gene effect was detected for ADHD symptom dimensions. Second, for executive function endophenotypes, positive correlations of the word interference time with hyperactive/impulsive and ADHD total scores were detected, likewise, the set‐shifting time and hyperactive/impulsive scores. Moreover, a complete mediation model was detected, showing that word interference time mediated the path between genotype and total scores; and the model suggests that the mechanism of SNP rs3908461 effects on ADHD total symptom was accounted by the indirect effect of inhibition control as the mediator. Third, for white matter architecture as the structural endophenotype, imaging genetic analyses showed significant associations between rs3908461 genotypes and FA values in corpus callosum, left superior longitudinal fasciculus, left posterior limb of internal capsule, left posterior thalamic radiate (include optic radiation), and the left anterior corona radiate.

Interestingly, our findings are in line with the recent research findings on lncRNAs, whose roles in neurodevelopmental disorders have been implicated by their involvements in a diverse range of neuronal functions, including neurogenesis, maintenance of pluripotency, synaptogenesis, nerve cells maturation, homeostasis, transcriptional regulation as well as synaptic plasticity and strength.^10^ A systematic analysis confirm that most of annotated lncRNAs in the human genome were exclusively expressed in the brain; some of these brain‐expressed lncRNAs are evolutionarily conserved and have the spatiotemporal expression specificity during the development of brain.^39^ Therefore, alterations in function of lncRNAs may contribute to the pathophysiology of several neurodevelopmental disorders including ADHD. These findings converge to support the biological plausibility of our findings.

Our first finding demonstrated that the allele distribution for *RNF219*‐*AS1* rs3908461 was significantly different between children with ADHD and controls. The SNP rs3908461 was located in the intron of a noncoding RNA gene of *RNF219*‐*AS1* (Ring Finger Protein 219 Antisense RNA 1) which is also regarded as ORC Ubiquitin Ligase 1 Antisense RNA 1(*OBI1*‐*AS1*) (www.genecards.org). *OBI1*‐*AS1* is a particularly interesting molecule in relation to ADHD. *OBI1*‐*AS1* molecule has been reported to be associated with several neuropsychiatric phenotypes/traits including smoking status, alcohol drinking, total ventricular volume in Alzheimer's disease, sleep duration, and antipsychotic drug‐induced weight gain in schizophrenia in the GWAS Catalog database (www.ebi.ac.uk/gwas/). A recent transcriptome‐wide association study (TWAS) based on ADHD GWAS has found that *RNF219*‐*AS1* was also associated with ADHD as genetically regulated gene.^40^ Although the exact function of *OBI1*‐*AS1* is still unclear, some studies suggest that natural antisense RNA hybridize with the endogenous mRNA to play the regulatory role.^41^ The host gene *OBI1 *has been found to be involved in the ubiquitin signaling pathway which regulated the DNA replication and effected cell growth and transformation.^42^ The ubiquitin‐proteasome system, regulating numerous cellular processes in eukaryotes including cell cycle and protein quality control, plays a pivotal role in the central nervous system and has linked to various psychiatry disorders such as SZ,^43^ ASD^44^ and depression.^45^ The dynamic change of the ubiquitination‐associated proteasome, from ubiquitination to deubiquitination, could affect the myelin proteins in cellular trafficking and the oligodendrocytes differentiation and are implicated in the demyelinating disease of central nervous system (CNS) such as multiple sclerosis.^46^ In addition, the ubiquitin signal could interact with autophagy and then cause the mitochondria damage and degradation.^47^ Hwang et al found that mitochondrial DNA haplogroups polymorphism was associated with ADHD children in Korean population.^48^ As such, how rs3908461 could affect ADHD expression through the specific molecular pathways deserve further exploration. While a recently published ADHD GWAS analyses identified two SNPs of *OBI1*‐*AS1*, rs2243638, and rs9574218 as ADHD‐related risk variants,^13^ the SNP rs3908461 identified in our sample nevertheless is independent from these two SNPs. The SNP rs3908461 did not show any linkage disequilibrium (LD) with those two SNPs neither in CHB nor CEU population (Figure S1). The evidence from eQTL analysis in brain also supported that SNP rs3908461 was functionally distinct from rs2243638 and rs9574218, in particular that rs3908461 could influence the *RNF219* expression in white matter while rs2243638 and rs9574218 in medulla (http://www.braineac.org/).

In our study, quantitative analyses detected nominal genetic effects of *RNF219*‐*AS1* rs3908461 on inhibition function deficits among ADHD subjects, with TT genotype (as protective factor) carriers performing better. Although no significant association was found for ADHD core symptoms, our mediation analysis found a significant indirect effect of *RNF219*‐*AS1* rs3908461 on ADHD total symptom through executive inhibition as the mediator. Previous studies have found that children with ADHD exhibited a range of executive deficits, especially the inhibition function.^49^ Family‐based behavioral genetics studies conducted in either ADHD^50^ or general population^35^ have suggested that inhibitory function—as an important executive function refers to the ability to control decision‐making, behaviors, and emotional impulse—is significantly heritable; and it may be considered a valid endophenotype of ADHD. Executive inhibition is also closely associated with other ADHD traits such as inattention and hyperactivity‐impulsivity; and shares substantial genetic risks. Twin studies showed that inhibitory defects in early childhood as a potential genetic risk factor predict later ADHD behavioral problems.^51^ To our knowledge, our study is the first to provide preliminary empirical support for the influence of lncRNA *RNF219*‐*AS1* on ADHD symptoms through inhibition function as the mediator. However, the detected association between rs3908461 and the inhibition in our study did not survive Bonferroni correction, and our findings must be regarded with caution and as preliminary, awaiting further replication.

The eQTL analyses based on the UK Brain Expression Cohort of Caucasian subjects showed that minor C‐allele increased the *RNF219* mRNA expression in white matter. *RNF219*‐*AS1* is a natural antisense transcript (NAT), whose transcript comes from the opposite strand of the adjacent protein‐coding gene *RNF219*. Such cis‐NAT can be partly or completely overlapping and bind to the sense strand and may regulate the activities of neighboring genes in the ways of transcription inhibition, nuclear RNA‐RNA interaction, RNA‐DNA interactions, and cytoplasmic RNA‐RNA interaction.^52^ The results form functional annotations of rs3908461 and its proxies (*r* ^2^ ≥ 0.8 in the 1000 Genomes, CHB population) in the bioinformatics database of HaploReg v.4.1 (https://pubs.broadinstitute.org/mammals/haploreg/haploreg.php) were shown in the Table S4, rs3908461 overlaps with the enhancer histone marks of embryonic stem cell‐derived neuron cultured cells (ESDR) and fat cells (FAT) and possible motifs to change transcription factor binding (Hoxb13, Hoxb9, and Nkx6‐1). These evidences support the potential transcriptional regulatory function of rs3908461. Given the information that *RNF219*‐*AS1* (chr13:78493824–79191463) and *RNF219* (chr13:79188421–79233314) were tail‐to‐tail overlaps with 3043 bp (https://genome.ucsc.edu/) aligned to the human reference genome (hg19), and rs3908461(chr13:78770857) was located in the second intron of *RNF219*‐*AS1*. We posited that the influence of rs3908461 on the expression of *RNF219* gene in the brain may be the part of the transcriptional regulatory mechanism for antisense lncRNA interacted with the host gene. However, we should note that the potential genetic effect on gene expression was only found when the exon‐specific probeset exprID3518981 was used, while not for the exprID t3518971 which was derived from the mean over 13 probesets. Such phenomenon has been found in some other studies.^53^, ^54^ The precise reason of the inconsistency between the overall gene expression and the individual exon‐specific probesets was not unclear based on the available data. One possible explanation is that the potential influence of genetic variants on the gene expression might be restricted to some specific transcripts, which might involve the complex process of gene splicing.^55^ More neurobiological studies were needed to explain. For our present study, the eQTL result was set as suggestive evidence for the following imaging genetic analysis to explore whether candidate SNPs could indeed affect the white matter features. The comprehensive relationship from “genetic variants” via “gene expression” to “brain structural alteration” could not be established at present. Future studies involving mRNA expression data of our own samples might promote such investigation.

To explore more fully the potential functional mechanisms of *OBI1*, we investigated the 11 key proteins which interacted with *OBI1* gene in the STRING Interaction Network (https://version11.string‐db.org/) (Figure 2) and found two of them associated with the degradation of myelin debris, the generation of new oligodendrocytes (*MYD88*),^56^ immune responses in CNS, and demyelinating disease activity (*TOB1*).^57^ As such, our FA findings of white matter architecture differences associated with C‐allele as structural endophenotype are in line with existing findings that implicate the roles of *OBI1* in myelination.

Our previous study has revealed that children with ADHD showed the decreased FA values in corpus callosum, the left superior longitudinal fasciculus, and the left corona radiate compared to the healthy.^38^ In present study, the rs3908461 genotypic main effects also identified differences in these same three white matter tracts. Moreover, we also detected significant correlation of inhibition and FA values in relation to three overlap regions (body of corpus callosum, left anterior corona radiate, and left superior longitudinal fasciculus) as the regions of interest (RIO). The decrease FA values reported in our previous study were related to impaired inhibition only in the children with ADHD in left superior longitudinal fasciculus. The superior longitudinal fasciculus (SLF) is a major frontoparietal white matter tract which connects the parietal and temporal brain regions with the frontal lobes^58^ and potentially plays an important role in complex cognitive processes including inhibitory control and set‐shifting.^59^ These are in line with findings from other studies. Wolfers et al also found that decreased FA values were associated with the poor attention in the right superior longitudinal fasciculus in adults with ADHD.^60^ The decreased FA might be an enduring trait of ADHD existing both in children and adults.^61^ Urger et al found that decreased FA values in the left SLF was also correlated with poor attention and language in health children and adolescents.^59^ It is possible that differential organization of white matter and the interactions between different brain regions may contribute to the functional diversity.^62^ Thus, the integrated analysis of genotype, behavior‐executive function and structural connectivity may be an effective approach to unravel the etiology of ADHD.

However, our current findings that risk‐allele carriers (C‐allele) were linked with increased FA values were not in line with our expectation. There could be three explanations. First functional changes could be more accurately determined by task‐based functional MRI studies^63^ rather than only white matter microstructure (FA values). Analyses of multi‐modal imaging data, including other brain structural features such as sulcal pits^64^ cerebral perfusion and functional brain connectivity,^65^ might help to elucidate the true direction of functional changes. Second, this counter‐intuitive finding, however, could represent a compensatory mechanism, similar to another study which found ADHD patients showed increased impulsivity and lower ventral striatal activity, yet the risk genotype carriers showed higher ventral striatal activity,^66^ which has been suggested to be a potential compensatory mechanism. As such, the increased FA values might be a secondary phenomenon in the disease progresses as a compensation for the decreased integrity of white matter upon the occurrence of ADHD. Finally, the increased FA values may reflect delayed neural pruning in our ADHD participants. Future replication studies are needed to confirm our findings or to unravel potential mechanisms.

Several limitations need be considered. First, for the functional endophenotype analysis, we only detected a marginal relationship between genetic variation and inhibitory function, and the result did not survive correction for the multiple comparisons. The correlation between inhibition and symptom score was also relatively weak. Our results should therefore be interpreted with caution. Second, the executive function endophenotypic analyses were conducted in the ADHD participants, and not in the controls, so the potential effects of lncRNA SNPs on executive function in the general population remained unexplored. Third, our sample included only participants of Chinese Han descent, then the generalizability of our findings to other ethnic groups and populations needs to be tested in the future. The genetic findings through the GWAS‐meta carried on adult/youth samples for the neurodevelopmental diseases may not be able to identify the genetic risk for childhood‐onset conditions.^67^ It would be appealing to enhance the inclusion of children for the research in psychiatry genetics with special attention to those of non‐European ancestry.^67^ We only recruited children samples in our present study that future studies are needed to verify whether the results are retained in the other age‐groups. Fourth, our present study was to investigate the association between lncRNAs genetic variants and ADHD by a post hoc analyses involving additional measurement. Replication in independent cohorts will verify and promote the interpretation of genetic effects of *RNF219*‐*AS1* in the etiology of ADHD. Lastly, we only focused on the effects of variations in the DNA polymorphisms of lncRNAs to detect the correlation with ADHD. We could not sequence the actual lncRNAs, which would necessitate accessing brain tissues and therefore would not be practically feasible in human studies. Moreover, we could not explore the molecular function of *RNF219*‐*AS1* with ADHD. Therefore, functional mechanisms could only be inferred. As an alternative strategy, in the future, we plan to analyze comprehensively the expression patterns of lncRNAs transcriptome of ADHD patients using high‐throughput sequencing technology to explore more fully the molecular mechanism by which lncRNAs influence ADHD etiology.

## 5 CONCLUSIONS

Our study explored the potential involvement of lncRNA *RNF219*‐*AS1* in the pathophysiology of ADHD at different phenotypic levels, levels guided by the Research Domain Criteria (RDoC) framework: (i) ADHD caseness and symptom dimension, (ii) executive functions as functional endophenotype, and (iii) potential genetic influence of rs3908461 in *RNF219*‐*AS1* on white matter architecture as brain structural endophenotype. LncRNAs, as the noncoding sequences widely expressed in the central nervous system, are likely to be involved in abroad spectrum of molecular functions in the brain development and thus of critical relevance to the pathogenesis of neuropsychiatric disorders included ADHD. Our findings provide preliminary support for an associations of lncRNAs in the complex pathogenesis of ADHD.

## CONFLICT OF INTEREST

The authors declare that the research was conducted in the absence of any commercial or financial relationships that could be construed as a potential conflict of interest.

## AUTHOR CONTRIBUTIONS

Guanghui Fu, Lu Liu, and Qiujin Qian contributed conception and design of the study; Haimei Li and Guanghui Fu organized the database; Guanghui Fu, Lu Liu, Wai Chen, Yufeng Wang, and Qiujin Qian performed the statistical analysis, interpreted the results, and wrote sections of the manuscript. All authors contributed to manuscript revision, read, and approved the submitted version.

## Supporting information

Supplementary Material

## Data Availability

The data that support the findings of this study are available from the corresponding author upon reasonable request.
