# Supplementary material for: A potential association of RNF219‐AS1 with ADHD: Evidence from categorical analysis of clinical phenotypes and from quantitative exploration of executive function and white matter microstructure endophenotypes
Source: CNS Neurosci Ther. 2021 Feb 28;27(5):603–16. doi: 10.1111/cns.13629 (PMC8025624; doi:10.1111/cns.13629)
Supplement: Supplementary file 1 — Supplementary Material [file CNS-27-603-s001.docx]

**Supplementary Materials**

**Supplementary Table 1 Allelic association between lncRNA SNPs and ADHD**

| **Gene Symbol** | **Gene region (hg19)** | **tag SNPs** | **Public location (hg19)** | **Major/minor allele** | **MAF** | **HWE** | **Call rate (%)** | **Risk Allele** | **Chi** | **p** |
| --- | --- | --- | --- | --- | --- | --- | --- | --- | --- | --- |
| *LINC02497* | chr4:31170766-  31215296 | rs1499464 | chr4:31177908 | C/G | 0.219 | 0.716 | 99.9 | G | 0.49 | 0.485 |
|  |  | rs6856841 | chr4:31188122 | G/A | 0.437 | 0.055 | 99.9 | A | 0.09 | 0.761 |
|  |  | rs2310325 | chr4:31193318 | T/A | 0.250 | 0.367 | 99.9 | T | 1.42 | 0.234 |
|  |  | rs9997440 | chr4:31208611 | A/T | 0.130 | 0.089 | 99.9 | A | 0.47 | 0.494 |
|  |  | rs7694638 | chr4:31212626 | T/C | 0.103 | 0.975 | 98.7 | T | 0.30 | 0.584 |
| *LINC00461* | chr5:87958263-  87974116 | rs6893807 | chr5:87965021 | T/C | 0.473 | 0.368 | 99.9 | C | 2.48 | 0.115 |
| *LINC02060* | chr5:87702799-  87736907 | rs12187346 | chr5:87720541 | A/G | 0.354 | 0.220 | 100 | A | 0 | 0.994 |
|  |  | rs12657135 | chr5:87725387 | T/C | 0.208 | 0.233 | 100 | T | 0.84 | 0.359 |
|  |  | rs4423262 | chr5:87734426 | A/G | 0.145 | 0.382 | 99.8 | G | 1.06 | 0.303 |
| *TMEM161B-AS1* | [chr5:87562841- 87734491](https://genome.ucsc.edu/cgi-bin/hgTracks?position=chr5:87564841-87732491&hgsid=740705957_yGWAsjA66aAV9MtfLyDU0ghMrePX&knownGene=pack&hgFind.matches=uc003kje.3,) | rs10514299 | chr5:87663610 | C/T | 0.144 | 0.247 | 99.6 | T | 1.31 | 0.253 |
|  |  | rs4916720 | chr5:87677848 | G/A | 0.363 | 0.452 | 99.9 | G | 0.14 | 0.711 |
|  |  | rs16903104 | chr5:87682987 | G/A | 0.210 | 0.325 | 99.6 | G | 1.65 | 0.199 |
| *LINC01288* | chr8:34639439-  34724316 | rs2687544 | chr8:34668196 | T/C | 0.146 | 0.384 | 98.6 | C | 2.11 | 0.146 |
|  |  | rs16883114 | chr8:34700530 | T/C | 0.439 | 0.804 | 99.6 | T | 1.19 | 0.276 |
| *LINC01572* | chr16:72315200-  72700908 | rs1946768 | chr16:72334370 | C/T | 0.127 | 0.073 | 97.3 | T | 1.16 | 0.282 |
|  |  | rs17607347 | chr16:72381460 | T/C | 0.337 | 0.423 | 99.2 | C | 1.01 | 0.314 |
|  |  | rs1080536 | chr16:72395453 | G/A | 0.157 | 0.015 | 97.5 | A | 0.27 | 0.603 |
|  |  | rs1946767 | chr16:72334538 | T/C | 0.400 | 0.028 | 100 | C | 2.57 | 0.109 |
|  |  | rs11862324 | chr16:72462749 | T/C | 0.496 | 0.992 | 100 | C | 2.58 | 0.109 |
|  |  | rs10492822 | chr16:72470984 | C/T | 0.268 | 0.760 | 100 | T | 0.79 | 0.375 |
|  |  | rs9630625 | chr16:72496681 | T/C | 0.499 | 0.932 | 100 | C | 1.56 | 0.212 |
|  |  | rs12596294 | chr16:72587093 | T/A | 0.170 | 0.664 | 100 | A | 0.72 | 0.398 |
|  |  | rs2860346 | chr16:72592309 | C/A | 0.405 | 0.825 | 100 | A | 0.74 | 0.391 |
|  |  | rs9935250 | chr16:72648087 | T/C | 0.236 | 0.901 | 100 | T | 5.59 | **0.018** |
|  |  | rs12924285 | chr16:72653326 | G/A | 0.135 | 0.781 | 100 | A | 0.36 | 0.547 |
| *MEF2C-AS1* | chr5:88177147-  88330196 | rs10044342 | chr5:88178683 | T/C | 0.129 | 0.815 | 99.5 | T | 0.44 | 0.509 |
|  |  | rs304141 | chr5:88188058 | G/A | 0.257 | 0.861 | 99.9 | C | 0.22 | 0.642 |
|  |  | rs4446500 | chr5:88196003 | A/G | 0.213 | 0.172 | 100 | A | 0.09 | 0.767 |
|  |  | rs304132 | chr5:88215594 | G/A | 0.279 | 0.163 | 100 | G | 0.12 | 0.726 |
|  |  | rs4916663 | chr5:88302330 | T/A | 0.356 | 0.746 | 99.8 | A | 0.27 | 0.603 |
|  |  | rs1427967 | chr5:88328566 | C/A | 0.436 | 0.914 | 99.9 | A | 1.79 | 0.181 |
| *LOC105379109* | chr5:104050055-  104111477 | rs11744448 | chr5:104052793 | C/T | 0.249 | 0.361 | 100 | C | 0.57 | 0.451 |
|  |  | rs324343 | chr5:104094519 | C/T | 0.212 | 0.706 | 97.1 | C | 0.01 | 0.908 |
|  |  | rs2304914 | chr5:104109619 | T/C | 0.467 | 0.321 | 99.9 | C | 0.18 | 0.675 |
| *RNF219-AS1* | chr13:78493824-  79191463 | rs7998775 | chr13:78522427 | C/T | 0.089 | 0.015 | 96.6 | T | 0.63 | 0.429 |
|  |  | rs9544656 | chr13:78550841 | C/T | 0.228 | 0.073 | 99.8 | T | 0.10 | 0.748 |
|  |  | rs1330896 | chr13:78603571 | G/C | 0.475 | 0.271 | 99.9 | C | 0.004 | 0.948 |
|  |  | rs944378 | chr13:78605893 | T/C | 0.109 | 1.000 | 100 | C | 0.01 | 0.914 |
|  |  | rs9544679 | chr13:78611819 | C/T | 0.368 | 0.45 | 99.6 | C | 0 | 0.988 |
|  |  | rs9318512 | chr13:78651560 | A/G | 0.472 | 0.910 | 98.0 | G | 1.81 | 0.179 |
|  |  | rs4144873 | chr13:78659208 | G/C | 0.493 | 0.012 | 97.7 | C | 0.60 | 0.437 |
|  |  | rs1854798 | chr13:78665761 | C/G | 0.187 | 0.164 | 99.9 | C | 0.23 | 0.633 |
|  |  | rs1556267 | chr13:78680420 | A/G | 0.074 | 0.614 | 99.9 | A | 0.11 | 0.745 |
|  |  | rs1330884 | chr13:78683509 | T/G | 0.470 | 0.370 | 99.0 | G | 2.70 | 0.100 |
|  |  | rs9530729 | chr13:78700751 | C/T | 0.256 | 0.127 | 96.4 | T | 1.42 | 0.233 |
|  |  | rs3861135 | chr13:78706449 | G/A | 0.299 | 0.519 | 100 | A | 1.28 | 0.258 |
|  |  | rs9600980 | chr13:78711714 | G/C | 0.395 | 0.545 | 99.8 | C | 4.29 | **0.038** |
|  |  | rs41381145 | chr13:78716024 | C/T | 0.171 | 0.685 | 100 | T | 0.54 | 0.461 |
|  |  | rs9544725 | chr13:78729367 | G/A | 0.198 | 0.474 | 98.5 | A | 0.40 | 0.528 |
|  |  | rs1324790 | chr13:78741934 | G/A | 0.065 | 1.000 | 100 | G | 1.53 | 0.217 |
|  |  | rs10507880 | chr13:78742120 | T/C | 0.217 | 0.459 | 100 | T | 5.42 | **0.020** |
|  |  | rs2763416 | chr13:78748745 | C/T | 0.254 | 0.393 | 99.7 | C | 7.94 | **4.80E-03** |
|  |  | rs2585565 | chr13:78750300 | G/A | 0.487 | 0.003 | 99.5 | G | 2.76 | 0.097 |
|  |  | rs3908461 | chr13:78770857 | C/T | 0.298 | 1.000 | 99.7 | C | 15.4 | **8.61E-05** |
|  |  | rs9601007 | chr13:78778897 | C/T | 0.207 | 1.000 | 99.9 | T | 3.37 | 0.066 |
|  |  | rs2038784 | chr13:78797009 | T/G | 0.477 | 0.069 | 99.8 | G | 2.03 | 0.155 |
|  |  | rs12583965 | chr13:78808813 | T/C | 0.126 | 0.668 | 99.9 | C | 0.11 | 0.746 |
|  |  | rs9574199 | chr13:78808914 | A/C | 0.301 | 0.040 | 97.2 | A | 0.21 | 0.646 |
|  |  | rs1410738 | chr13:78813179 | G/T | 0.202 | 0.151 | 98.1 | G | 2.75 | 0.097 |
|  |  | rs9565397 | chr13:78814720 | T/C | 0.369 | 0.007 | 97.7 | T | 1.48 | 0.223 |
|  |  | rs3949946 | chr13:78820358 | C/A | 0.375 | 0.125 | 96.2 | A | 1.72 | 0.190 |
|  |  | rs1972227 | chr13:78820747 | C/T | 0.104 | 0.701 | 99.8 | C | 0.08 | 0.779 |
|  |  | rs9565398 | chr13:78833871 | G/A | 0.452 | 0.349 | 97.5 | G | 0.20 | 0.654 |
|  |  | rs4884088 | chr13:78847455 | C/T | 0.346 | 0.399 | 99.1 | T | 0.01 | 0.907 |
|  |  | rs9601024 | chr13:78850176 | C/T | 0.160 | 0.218 | 99.2 | C | 0.13 | 0.717 |
|  |  | rs9530773 | chr13:78852243 | A/C | 0.064 | 0.748 | 99.9 | A | 0.03 | 0.859 |
|  |  | r17069491 | chr13:78856681 | C/T | 0.132 | 0.013 | 99.0 | T | 0.39 | 0.531 |
|  |  | rs1324794 | chr13:78861536 | T/C | 0.196 | 0.033 | 96.7 | C | 0.11 | 0.738 |
|  |  | rs988016 | chr13:78904481 | C/A | 0.343 | 0.474 | 99.0 | C | 0.01 | 0.911 |
|  |  | rs9544774 | chr13:78905831 | G/A | 0.115 | 0.784 | 97.3 | A | 0.41 | 0.523 |
|  |  | rs11619660 | chr13:78920066 | A/C | 0.155 | 0.819 | 99.2 | A | 0.76 | 0.383 |
|  |  | rs9574221 | chr13:78925775 | A/G | 0.260 | 0.127 | 100 | G | 0.97 | 0.326 |
|  |  | rs1967453 | chr13:78931663 | G/A | 0.115 | 0.727 | 99.9 | G | 0.12 | 0.733 |
|  |  | rs9544780 | chr13:78952444 | A/G | 0.122 | 0.265 | 100 | G | 0.45 | 0.501 |
|  |  | rs17069694 | chr13:78955933 | C/T | 0.137 | 0.598 | 99.7 | T | 0.29 | 0.591 |
|  |  | rs9318541 | chr13:78958248 | T/C | 0.401 | 0.509 | 99.3 | T | 0.03 | 0.854 |
|  |  | rs9574228 | chr13:78970629 | G/A | 0.362 | 0.189 | 99.9 | A | 0.25 | 0.614 |
|  |  | rs2876725 | chr13:78971895 | A/G | 0.283 | 0.513 | 99.9 | G | 0.64 | 0.424 |
|  |  | rs9318544 | chr13:78973603 | C/T | 0.254 | 0.125 | 100 | T | 2.57 | 0.109 |
|  |  | rs12860641 | chr13:78975155 | C/T | 0.143 | 0.930 | 100 | C | 0.61 | 0.434 |
|  |  | rs9601035 | chr13:78975771 | C/A | 0.325 | 0.651 | 98.9 | C | 0.17 | 0.678 |
|  |  | rs9530781 | chr13:78979249 | G/A | 0.083 | 0.251 | 95.1 | G | 1.15 | 0.284 |
|  |  | rs9565410 | chr13:78980541 | C/T | 0.481 | 0.157 | 100 | T | 1.96 | 0.161 |
|  |  | rs4147057 | chr13:78981117 | C/A | 0.212 | 0.213 | 99.7 | C | 0.17 | 0.677 |
|  |  | rs17069808 | chr13:78981493 | T/C | 0.228 | 0.338 | 100 | C | 0.05 | 0.818 |
|  |  | rs2329077 | chr13:78982173 | C/T | 0.262 | 0.636 | 99.8 | C | 0.27 | 0.603 |
|  |  | rs7338176 | chr13:78986898 | C/T | 0.439 | 0.544 | 100 | C | 1.16 | 0.282 |
|  |  | rs10507887 | chr13:78987956 | G/A | 0.178 | 0.738 | 100 | G | 0.60 | 0.439 |
|  |  | rs11619850 | chr13:79009488 | C/A | 0.200 | 0.443 | 100 | A | 0.003 | 0.956 |
|  |  | rs9318550 | chr13:79015424 | T/C | 0.376 | 0.371 | 99.5 | T | 0.03 | 0.859 |
|  |  | rs2329089 | chr13:79015537 | A/T | 0.203 | 0.532 | 99.5 | T | 0.33 | 0.568 |
|  |  | rs9593324 | chr13:79016232 | T/C | 0.194 | 0.993 | 99.8 | C | 0.03 | 0.874 |
|  |  | rs17069885 | chr13:79019147 | A/G | 0.274 | 0.023 | 99.0 | G | 0.09 | 0.770 |
|  |  | rs2329090 | chr13:79019949 | C/T | 0.347 | 0.366 | 99.9 | T | 0.06 | 0.800 |
|  |  | rs9318551 | chr13:79024308 | G/A | 0.169 | 1.000 | 98.6 | A | 0.003 | 0.953 |
|  |  | rs17069905 | chr13:79037359 | A/G | 0.484 | 0.121 | 99.6 | A | 0.10 | 0.752 |
|  |  | rs7337666 | chr13:79073248 | A/C | 0.437 | 0.254 | 100 | A | 0.35 | 0.554 |
|  |  | rs1985801 | chr13:79076111 | T/C | 0.368 | 0.519 | 100 | T | 0.08 | 0.782 |
|  |  | rs2876726 | chr13:79086353 | T/C | 0.052 | 0.589 | 98.7 | T | 0.01 | 0.931 |
|  |  | rs9574262 | chr13:79106435 | A/G | 0.460 | 0.417 | 99.9 | A | 0.24 | 0.625 |
|  |  | rs7328541 | chr13:79107402 | G/A | 0.224 | 0.655 | 99.3 | G | 0.01 | 0.939 |
|  |  | rs12873473 | chr13:79114661 | C/T | 0.097 | 0.234 | 99.8 | T | 0.36 | 0.551 |
|  |  | rs9593339 | chr13:79114971 | T/C | 0.147 | 0.474 | 100 | C | 0.24 | 0.625 |
|  |  | rs883494 | chr13:79132519 | T/C | 0.366 | 0.602 | 99.9 | C | 0.10 | 0.752 |
|  |  | rs2806593 | chr13:79153347 | A/T | 0.184 | 0.949 | 100 | A | 0.51 | 0.475 |
|  |  | rs9601084 | chr13:79160879 | G/T | 0.172 | 0.706 | 99.8 | T | 0.40 | 0.525 |
|  |  | rs9601097 | chr13:79181332 | C/G | 0.306 | 0.693 | 100 | G | 0.16 | 0.689 |

Note. The table shows 107 tag SNPs from 9 lncRNAs for genetic analyses in this study. SNP, single nucleotide polymorphism; MAF, minor allele frequency; HWE, Hardy–Weinberg equilibrium; the nominally significant results (*P* <0.05, uncorrected) were shown in **bold**.

**Supplementary Table 2** **Correlations between the Executive functions and ADHD core symptoms**

| **Executive function** | | **Inattentive scores** | **Hyperactive-Impulsive scores** | **Total scores** |
| --- | --- | --- | --- | --- |
|  |  | **r(*P*)** | **r(*P*)** | **r(*P*)** |
| **STROOP Color–Word Interference test** | **color interference time** | 0.01(0.698) | 0.04 (0.324) | 0.03(0.376) |
|  | **word interference time** | 0.06(0.095) | 0.13(**4.460E-4**) | 0.12(**0.001**) |
| **Trail making test (TMT)** | **set-shifting time** | 0.01(0.790) | 0.08(**0.024**) | 0.07(0.064) |
| **Adjusted with covariates**^a^ |  |  |  |  |
|  | **word interference time** |  | 0.07(0.058) | 0.08(**0.022**) |
|  | **set-shifting time** |  | 0.01(0.732) |  |

Note. ^a^ Adjusted with sex, age and IQ; the nominally significant results (*P* <0.05, uncorrected) were shown in **bold**.

**Supplementary Table 3** **Mediation of Word Interference time on the relationship between rs3908461 and ADHD total scores in children with ADHD^a^**

| **Variables** | **Word Interference time (M)** | | | | |  | **Total scores (Y)** | | | | | |
| --- | --- | --- | --- | --- | --- | --- | --- | --- | --- | --- | --- | --- |
|  | **β** | **SE** | **t** | **95%CI^b^** | |  | **β** | **SE** | **t** | | **95%CI^b^** | |
|  |  |  |  | **LLCI** | **ULCI** |  |  |  |  |  | **LLCI** | **ULCI** |
| **constant** | 63.02 | 7.54 | 8.36*** | 48.22 | 77.82 |  | 38.59 | 3.66 | 10.55*** | | 31.41 | 45.78 |
| **rs3908461(X)** | 5.49 | 2.49 | 2.20* | 0.60 | 10.38 |  | -0.11 | 1.16 | -0.10 | | -2.40 | 2.17 |
| **Word Interference time(M)** |  |  |  |  | |  | 0.04 | 0.02 | 2.28* | | 0.01 | 0.07 |
| **R^2^** | 0.19 |  |  |  | |  | 0.03 |  |  | |  | |
| **F** | 44.33*** |  |  |  | |  | 4.16** |  |  | |  | |
| **Mediation**  **(Indirect effect of X on Y)** |  |  |  |  | |  | **β** | **BootSE** | | **95%CI^b^** | | |
|  |  |  |  |  | |  |  |  |  | **LLCI** | | **ULCI** |
|  |  |  |  |  | |  | 0.21 | 0.12 | | 0.02 | | 0.50 |

Note. X: independent variable; Y: dependent variable; M: mediator; 95% CI: 95% confidence interval; LLCI: lower limit confidence interval; ULCI: upper limit confidence interval.* p <0.05, ** p <0.01, ***p<0.001.

^a^ Adjusted with sex, age and IQ; ^b^ Effects are significant when the upper and lower bound of the bias corrected 95% confidence intervals (CI) does not contain zero.

**Supplementary Table 4** **Functional annotation of rs3908461 and its proxies (r^2^≥0.8)**

| **variant** | **GERP cons** | **SiPhy cons** | **Enhancer histone marks** | **DNAse** | **Motifs changed** | **GRASP**  **QTL hits** | **GeneCard/**  **UCSC** | **dbSNP func annot** |
| --- | --- | --- | --- | --- | --- | --- | --- | --- |
| [rs7983212](https://pubs.broadinstitute.org/mammals/haploreg/detail_v4.1.php?query=&id=rs7983212) |  |  | ESDR | ESDR |  |  | *RNF219-AS1* | intronic |
| [rs1998657](https://pubs.broadinstitute.org/mammals/haploreg/detail_v4.1.php?query=&id=rs1998657) |  |  |  |  | Dobox4,Evi-1,GR |  | *RNF219-AS1* | intronic |
| [rs2255419](https://pubs.broadinstitute.org/mammals/haploreg/detail_v4.1.php?query=&id=rs2255419) |  |  |  |  | NRSF,Sox |  | *RNF219-AS1* | intronic |
| [rs2763419](https://pubs.broadinstitute.org/mammals/haploreg/detail_v4.1.php?query=&id=rs2763419) |  |  |  |  | 4 altered motifs | 1 hit | *RNF219-AS1* | intronic |
| [rs2038786](https://pubs.broadinstitute.org/mammals/haploreg/detail_v4.1.php?query=&id=rs2038786) |  |  | FAT, MUS, LNG |  | 7 altered motifs |  | *RNF219-AS1* | intronic |
| [rs7326174](https://pubs.broadinstitute.org/mammals/haploreg/detail_v4.1.php?query=&id=rs7326174) |  |  | 5 tissues |  | CEBPB,p300 |  | *RNF219-AS1* | intronic |
| [rs7326598](https://pubs.broadinstitute.org/mammals/haploreg/detail_v4.1.php?query=&id=rs7326598) |  |  | 5 tissues | BRN | 5 altered motifs |  | *RNF219-AS1* | intronic |
| [rs3908461](https://pubs.broadinstitute.org/mammals/haploreg/detail_v4.1.php?query=&id=rs3908461) |  |  | ESDR, FAT |  | Hoxb13,Hoxb9,Nkx6-1 |  | *RNF219-AS1* | intronic |
| [rs68101384](https://pubs.broadinstitute.org/mammals/haploreg/detail_v4.1.php?query=&id=rs68101384) |  |  | ESDR |  | Evi-1,GATA,SIX5 |  | *RNF219-AS1* | intronic |
| [rs1555711](https://pubs.broadinstitute.org/mammals/haploreg/detail_v4.1.php?query=&id=rs1555711) |  |  |  |  | Hbp1,RXRA |  | *RNF219-AS1* | intronic |
| [rs8000437](https://pubs.broadinstitute.org/mammals/haploreg/detail_v4.1.php?query=&id=rs8000437) |  |  |  |  | Hoxb13,Hoxb9,SIX5 |  | *RNF219-AS1* | intronic |
| [rs2038785](https://pubs.broadinstitute.org/mammals/haploreg/detail_v4.1.php?query=&id=rs2038785) |  |  | ESDR |  | 15 altered motifs |  | *RNF219-AS1* | intronic |
| [rs2147485](https://pubs.broadinstitute.org/mammals/haploreg/detail_v4.1.php?query=&id=rs2147485) |  |  |  |  | HNF1,Hoxa5,Mef2 |  | *RNF219-AS1* | intronic |
| [rs1555709](https://pubs.broadinstitute.org/mammals/haploreg/detail_v4.1.php?query=&id=rs1555709) |  |  |  |  | GATA,Sox |  | *RNF219-AS1* | intronic |
| [rs3903892](https://pubs.broadinstitute.org/mammals/haploreg/detail_v4.1.php?query=&id=rs3903892) |  |  |  | BRN |  |  | *RNF219-AS1* | intronic |
| [rs9318528](https://pubs.broadinstitute.org/mammals/haploreg/detail_v4.1.php?query=&id=rs9318528) |  |  |  |  | E2A,TATA |  | *RNF219-AS1* | intronic |
| [rs9544752](https://pubs.broadinstitute.org/mammals/haploreg/detail_v4.1.php?query=&id=rs9544752) |  |  |  |  | 4 altered motifs |  | *RNF219-AS1* | intronic |
| [rs9530766](https://pubs.broadinstitute.org/mammals/haploreg/detail_v4.1.php?query=&id=rs9530766) |  |  |  |  | 8 altered motifs |  | *RNF219-AS1* | intronic |

Note. GERP cons conserved site identified by GERP and SiPhy cons conserved site identified by SiPhy, Enhancer histone marks cell types where SNPs are in histone marks of enhancer, DNase cell types where SNPs are in the accessible region of DNase.

**Supplementary Figure 1** Linkage disequilibrium (*r*^2^) structure of rs3908461, rs2243517 and rs9574218 of *RNF219-AS1* calculated in the CHB (**a**) and CEU (**b**) population separately, based on the data from the International HapMap Project database (http://www.hapmap.org) and using HaploView 4.2 software. Numbers into the squares indicate R-squared; CHB, *Chinese* Han Beijing population; CEU, Utah residents with northern and western European ancestry.

**
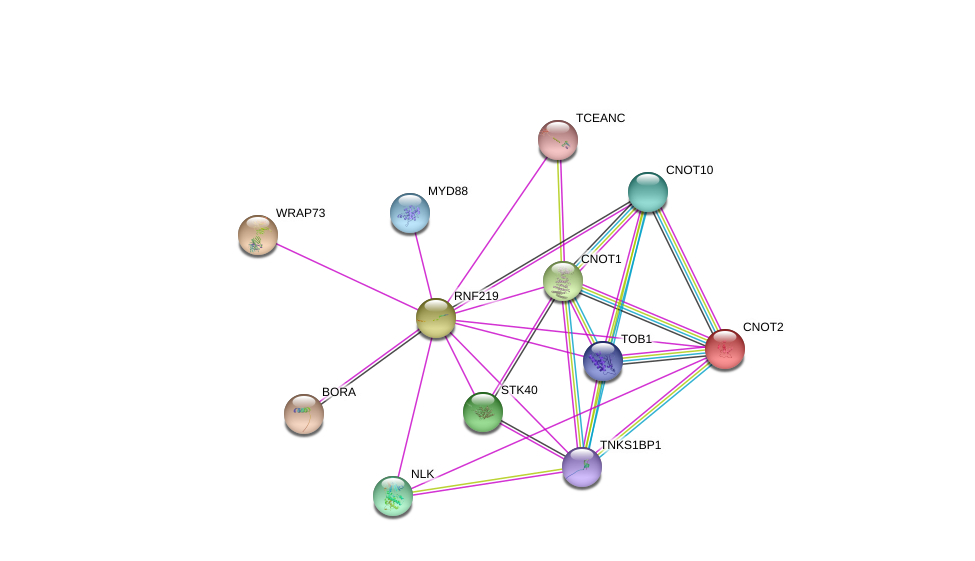
Supplementary Figure 2** The protein interacting network for *RNF219* (showing top 11 interacted genes) (<https://version11.string-db.org/>)
